# Immune checkpoint inhibitor (ICI)-based treatment beyond progression with prior immunotherapy in patients with driver-gene negative advanced non-small cell lung cancer

**DOI:** 10.1186/s12885-024-12315-5

**Published:** 2024-05-07

**Authors:** Min Wang, Xuquan Jing, Feihu Chen, Shuangqing Lu, Yulan Sun

**Affiliations:** 1 Department of Radiation Oncology, Shandong Cancer Hospital and Institute, Shandong First Medical University, Shandong Academy of Medical Sciences, Jinan, 250117 Shandong Province China; 2 Department of Medical Oncology, Shandong First Medical University and Shandong Academy of Medical Sciences, Shandong Cancer Hospital and Institute, 440 Jiyan Road, Jinan, 250117 Shandong Province China

**Keywords:** NSCLC, Immunotherapy, Progrssion, Cycles, Best response

## Abstract

**Background:**

No definite conclusion has yet to be reached for immunotherapy beyond progression(IBP) of first-line immunotherapy as the second-line treatment for advanced NSCLC patients with negative driver genes. Therefore a retrospective study was conducted to evaluate the efficacy of IBP in this population and investigated whether the cycles best response and progressive mode of first-line immunotherapy could affect the results.

**Patients and methods:**

The clinical data of patients with advanced NSCLC whose response was evaluated as progressive disease (PD) after receiving a PD-1/PD-L1 inhibitors as first-line therapy were retrospectively collected and the patients were assigned to the IBP and non-IBP groups. The overall survival (OS), progression-free survival (PFS) were evaluated between the two groups. The survival effects of cycles best response and progressive mode of first-line immunotherapy were also evaluated.

**Results:**

Between January 2019 and January 2022, a total of 121 patients was evaluated as PD after first-line immunotherapy in our institution; 53 (43.8%) patients were included in the IBP group and 68 (56.2%) patients were included in the non-IBP group. The OS and PFS were no significantly different between the two groups in whole population. Further analysis revealed the OS was prolonged with the prolongation of first-line medication cycle. The median OS was 15.4m (15.4 vs 10.8 *p*=0.047) 16.1m (16.1 vs 10.8 *p*=0.039), 16.3m (16.3 vs 10.9 *p*=0.029) for patients with ≥4, ≥6, ≥8 cycles in first-line immunotherapy, respectively. The advantages of OS and PFS were also seen in the subgroup of PR (best response) and oligo progression of first-line immunotherapy.

**Conclusions:**

The clinical outcomes of IBP were similar to those of non-IBP in patients with PD after first-line immnuotherapy in advanced NSCLC. But more cycles, PR as best response and oligo progression in first-line was benefit.

## Introduction

According to the Global Cancer Statistics 2020, lung cancer is remained the leading cause of cancer death [1]. Among them, patients with non–small-cell lung cancer (NSCLC) pathological type accounts for 85%, while the 5-year survival rate was less than 16% [2]. Therefore, there is an urgent need for improving the survival of patients with advanced non-small cell lung cancer (aNSCLC).

Starting from the clinical development of second-line monotherapy, Immune checkpoint inhibitors (ICIs), including anti-programmed cell death-1 (PD-1) and anti-programmed cell death ligand-1 (PD-L1) antibodies, have contributed greatly to improving the survival rate to driver gene-negative aNSCLC patients, no matter in first-line or second-line therapy [2–8]. However, there is no definite conclusion has yet to be reached for whether ICIs benefit patients beyond progression (IBP) of first-line immunotherapy as the second-line treatment for aNSCLC patients. According to post-hoc analyses of Keynote 010 study, 14 patients were retreated with Pembrolizumab after PD and achieved an ORR of 42.9% and a DCR of 78.6% [9]. And 51% of atezolizumab-arm patients who developed PD in OAK study continued to receive atezolizumab as treatment beyond progression (TBP). OS in TBP group was longer than switched to non-protocol anti-cancer therapy and no follow-up anti-tumor treatment (12.7 months vs. 8.8 vs, 2.2 months) [10]. These studies suggested that re-receiving PD-1 inhibitors still a possible benefit when tumors progress.

In this context, we conducted this retrospectively study under to investigate the effective of IBP and non-IBP treatment in aNSCLC patients. Furthermore, we evaluated whether the cycles, best response and progressive mode of first-line immunotherapy could affect the results.

## Materials and methods

### Patients

We retrospectively screened the records of advanced NSCLC patients whose response was evaluated as progressive disease (PD) after receiving a PD-1/PD-L1 inhibitors as first-line therapy in Shandong Cancer Hospital and Institute (Jinan, Shandong, China) between January 2019 and January 2022. The inclusion criteria were: (1) Eastern Cooperative Oncology Group performance status (ECOG PS) score of 0-1; (2) NSCLC confirmed by pathological or cytologically diagnosis; (3) stage IV or recurrent disease according to the eighth edition of the TNM classification for lung cancer; (4) received at least two cycles of ICIs in first-line treatment; (5) confirmed PD after first-line therapy using radiological examinations including chest computed tomography (CT), positron emission tomography (PET), magnetic resonance imaging (MRI), bone scan, ultrasound examination, or CT of the abdomen. The exclusion criteria were: (1) EGFR mutations or ALK/ROS1 rearrangements detected by amplification refractory mutation system polymerase chain reaction (ARMS-PCR) or next generation sequencing (NGS); (2) Patients with multiple primary tumors; (3) without progression or loss of follow-up in first-line therapy. Anonymized clinical data were collected from medical records, including gender, age, smoking status, histological subtype, gene alteration status, PD-L1 expression status, ECOG PS score, liver/brain metastases, best response to the first-line, progression mode of first-line, first/second-line therapy regimen.

### Treatment

Patients who were treated with ICIs for more than 2 cycles after PD were defined as IBP, while those who received ICI treatment for less than 2 cycles or discontinued it due to the PD were defined as non-IBP.

### Assessment of response

The response evaluation of tumors was based on the Response Evaluation Criteria in Solid Tumors (RECIST) version 1.1. evaluation was performed routinely every 6–8 weeks after starting treatment with the PD-1/PD-L1 inhibitor. Adverse events (AEs) were assessed according to the National Cancer Institute Common Terminology Criteria for Adverse Events (CTCAE) version 4.0. AEs that occurred during hospitalization were registered and graded by their attending doctor timely, while AEs that occurred outside the hospital were mainly based on patients’ initiative report. In our study, oligo progression was defined as ≤ 2 sites and ≤ 2 lesions of progression. Extensive progression was defined as≥ 3 sites and ≥ 3 lesions of progression.

### Endpoints

The primary study objective was overall survival (OS), defined as the time from the initiation of the post-PD treatment to death from any cause. The secondary objectives were progression-free survival (PFS). PFS was defined as the time from the initiation of the post-PD treatment to disease progression or death from any cause, whichever came first. The date of the last follow-up was October 1,2022 and the follow-up rate was 92.6%.

### Statistical analysis

All statistical analyses were performed using GraphPad Prism software version 8.0 (GraphPad Software, Inc., United States) and SPSS statistical software version 20.0 (IBM Corp., United States). The comparisons of patients’ baseline characteristics, tumor response in the two groups were analyzed using the Chi-square test and Fisher’s exact test. Univariate survival analysis was performed using the Kaplan–Meier method. Multivariate survival analysis was performed by a Cox proportional hazards model to evaluate the independent prognostic factors associated with improved survival. The Kaplan–Meier method was used to calculate OS and PFS. The difference in survival curves between the two groups was estimated by the log-rank test. Two-sided P values < 0.05 were considered statistically significant.

## Results

### Patients characteristics

**Fig. 1:**
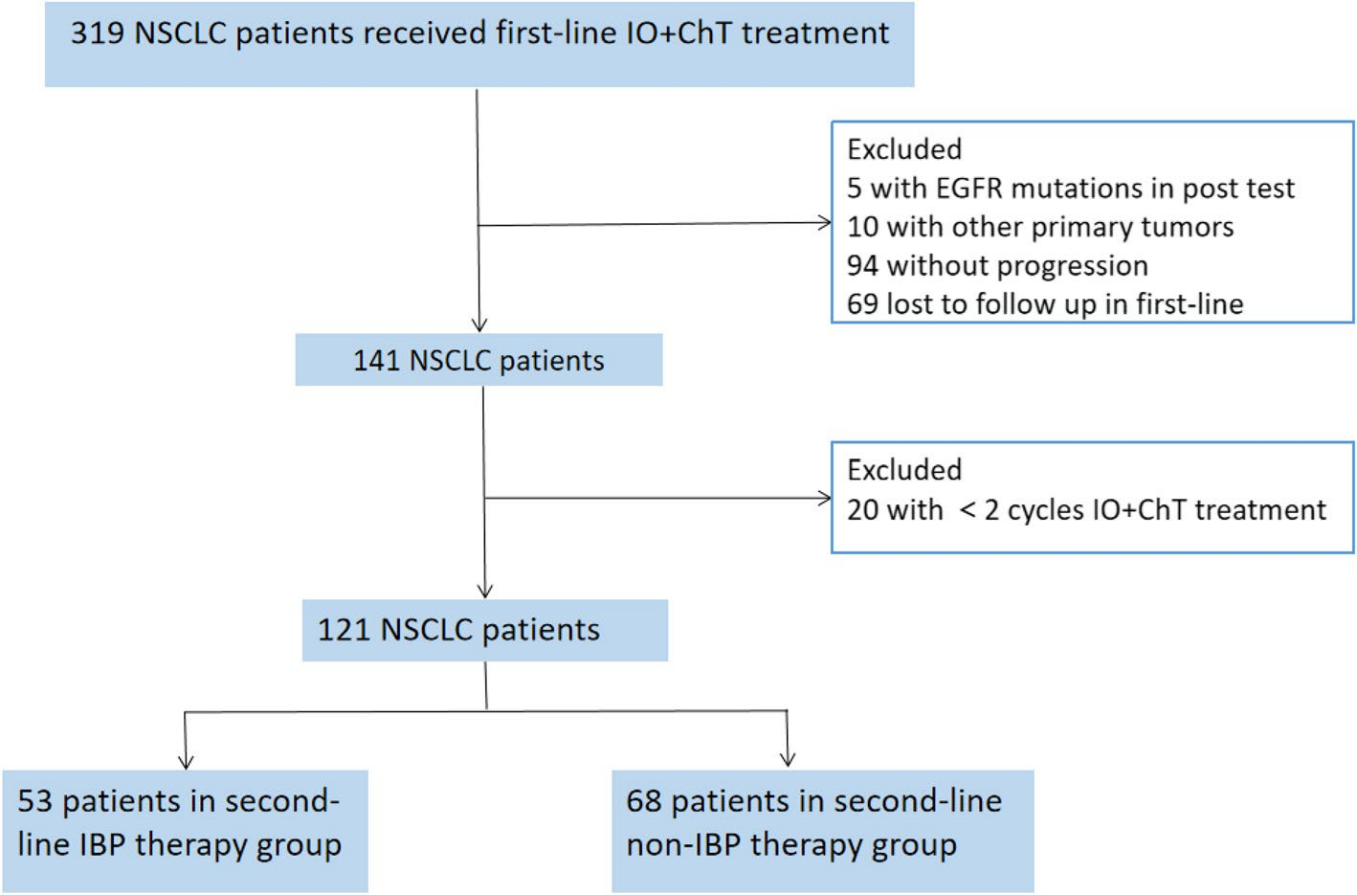
Study diagram

Between January 2019 and January 2022, 319 patients received immunotherapy in our institution. 94 patients were excluded for without progression, and 69 patients were excluded for lost to follow up in first-line. 10 patients were excluded for with other primary tumors, and 5 patients were excluded for with EGFR mutations in post-test. 141 aNSCLC patients was finally included due to treated with ICIs in second-line after immunotherapy PD in first-line. Then 20 patients were excluded for with <2 cycles IO+ChT treatment in first-line. As a result, a total of 121 patients who received PD-1/PD-L1 inhibitor as the second-line or later therapy were enrolled in the study (Fig. 1). According to the therapeutic modality, there were 53 patients in the IBP group and 68 patients in the non-IBP group. The last follow-up time was October 1, 2022. 48 patients had died by the end of the follow-up, and 73 patients were still alive. The median follow-up time was 5.6 months (range, 0.2– 29.0 months) for surviving patients and 13.1 months (range, 0.2–29.0 months) for all patients. The baseline characteristics of all patients in both groups are shown in Table 1. There were no differences between the two groups in the distribution of most variables except for treatment features. The median age of the patients in the two groups was 62 years, and the age range was 27 to 76 years. Fifty- three (43.8%) patients received IBP treatment, and 68 (56.2%) patients received non-IBP treatment. Eigthy-seven (71.9%) patients have adenocarcinoma pathology, 34 (28.1%) patients have squamous pathology. Thirty-three (28.1%) patients had brain metastases, and 20 (16.5%) patients had liver metastases. Sixty-four (52.9%) patients had never smoked. Fifty-four patients underwent PD- L1 detection: 17 (14.0%) patients had a level <1%, 19 (15.7%) patients had a level from 1 to 49%, and 18 (14.9%) patients had a level ≥ 50% (Table 1). The loss of follow-up rate was 7.4%.

**Table 1:** Clinical features

Characteristic	Total	IBP		non-IBP		*p*
		No.	%	No.	%	
Gender						
Male	86	38	71.7%	48	70.6%	
Female	35	15	28.3%	20	29.4%	0.894
Age						
<65	74	31	58.5%	43	63.2%	
≥65	47	22	41.5%	25	36.8%	0.595
Smoker						
Yes	57	26	49.1%	31	45.6%	
No	64	27	50.9%	37	54.4%	0.705
Smoking pack-years						
0	64	27	50.9%	37	54.4%	
>0-10	11	5	9.4%	6	8.8%	
>10-20	10	4	7.5%	6	8.8%	
>20-30	15	8	15.1%	7	10.3%	
>30-40	8	3	6.0%	5	7.4%	
>40-50	5	2	3.8%	3	4.4%	
>50	8	4	7.5%	4	5.9%	
Histology						
adenomatous	87	36	67.9%	51	75.0%	
squamous	34	17	32.1%	17	25.0%	0.390
Brain Meta						
Yes	34	16	30.2%	18	26.5%	
No	87	37	69.8%	50	73.5%	0.652
Liver Meta						
Yes	20	10	18.9%	10	14.7%	
No	101	43	81.1%	58	85.3%	0.541
PD-L1 status						
<1%	17	5	9.4%	12	17.6%	
1-49%	19	12	22.6%	7	10.3%	
≥50%	18	12	22.6%	6	8.8%	
No examined	67	24	45.3%	43	63.2%	0.020
First-line cycles of immunotherapy						
<4	12	7	13.2%	5	7.4%	
4-7	42	17	32.1%	25	36.8%	
≥8	67	29	54.7%	38	55.9%	0.542
Best response to first line						
PR	48	20	37.7%	28	41.2%	
SD	63	27	50.9%	36	52.9%	
PD	10	6	11.3%	4	5.9%	0.555
Progressive mode						
Oligo progression	57	26	49.1%	31	45.6%	
Extensive progression	64	27	50.9%	37	54.4%	0.705
Second-line therapy						
IO+ChT+A	20	20	37.7%			
IO+ChT	17	17	32.1%			
IO+A	14	34	26.4%			
IO	2	2	3.8%			
A+ChT	26			26	38.2%	
A	7			7	10.3%	
ChT	15			135	22.1%	
Other therapy	20			20	29.4%	

### Survival

**Fig. 2:**
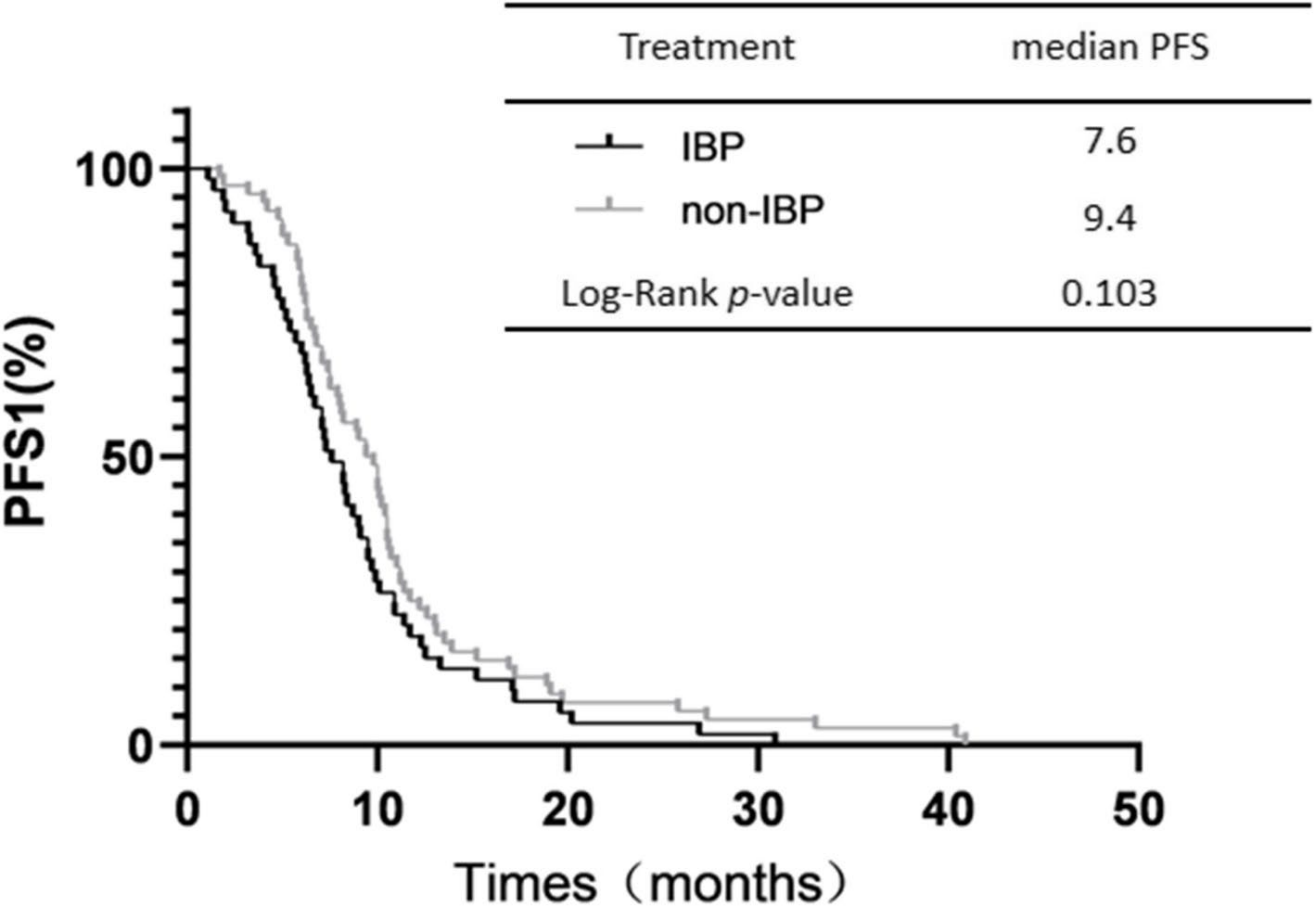
IBP versus non-IBP in second-line treatment of PFS1 in advanced NSCLC patients. Abbreviations: PFS, progression-free survival

**Fig. 3:**
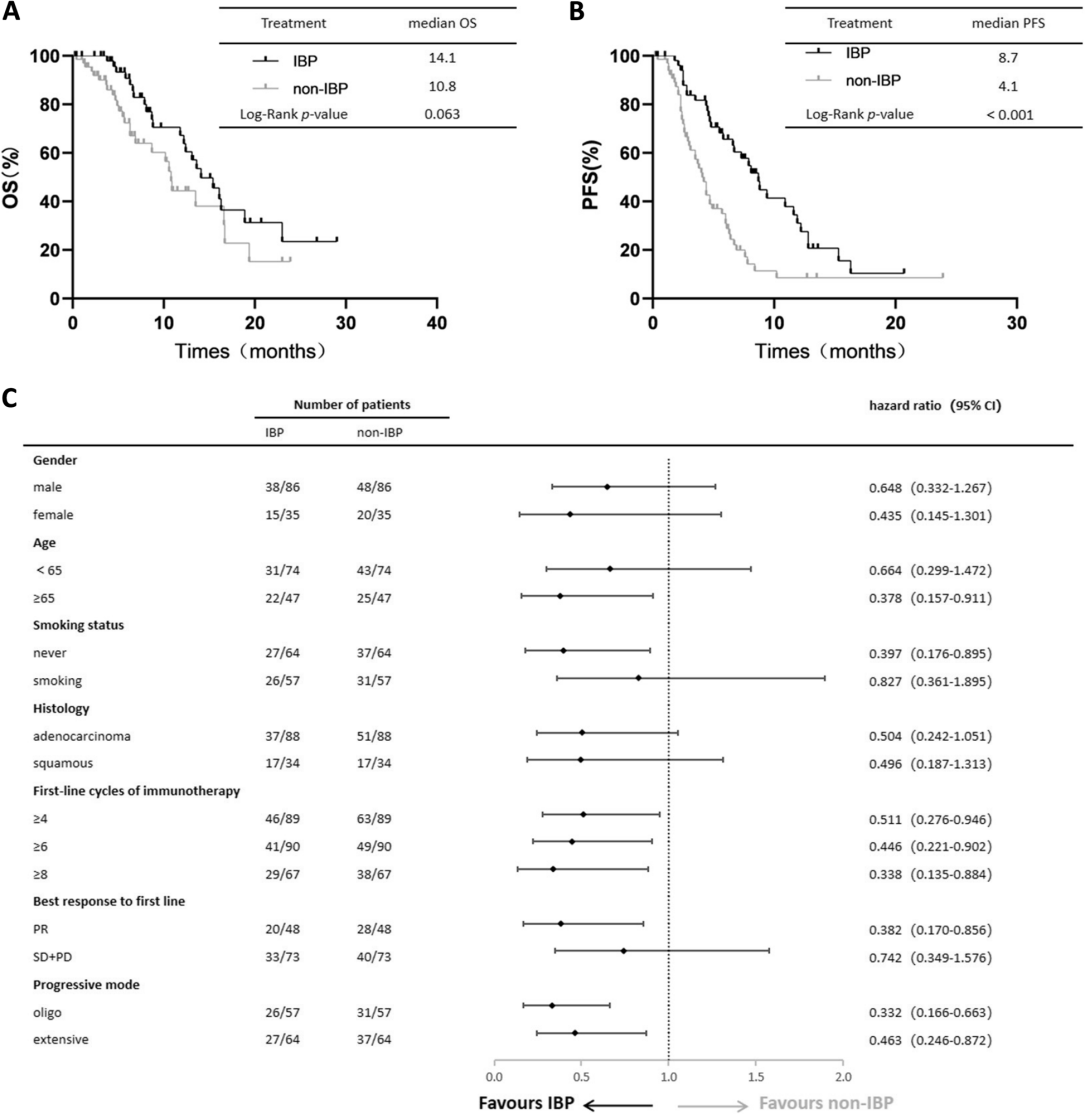
IBP versus non-IBP in second-line treatment of OS (**A**) and PFS (**B**) in advanced NSCLC patients. Subgroup analysis of OS (**C**). Abbreviations: IBP, immunotherapy beyond progression; NSCLC, non-small-cell lung cancer; OS, overall survival

The median PFS1 in the IBP group and non-IBP group in first-line therapy was 7.6 and 9.4 months, respectively. PFS1 was not significantly different between the two groups (*p*=0.103 Fig. 2). The median OS in the IBP group and non-IBP group was 14.1 and 10.8 months, and not statistically difference (*p*=0.063 Fig. 3A). The OS rates at 1 year in the IBP group and non-IBP group were 37.7% and 13.2%, respectively. The median PFS was 8.7 months for the patients receiving IBP treatment and 4.1 months for those receiving non-IBP treatment in second-line therapy. The PFS was longer in the IBP group than in the non-IBP group (*p* < 0.001, Fig. 3B). Partial subgroup analysis of OS was in Fig. 3C.

**Fig. 4:**
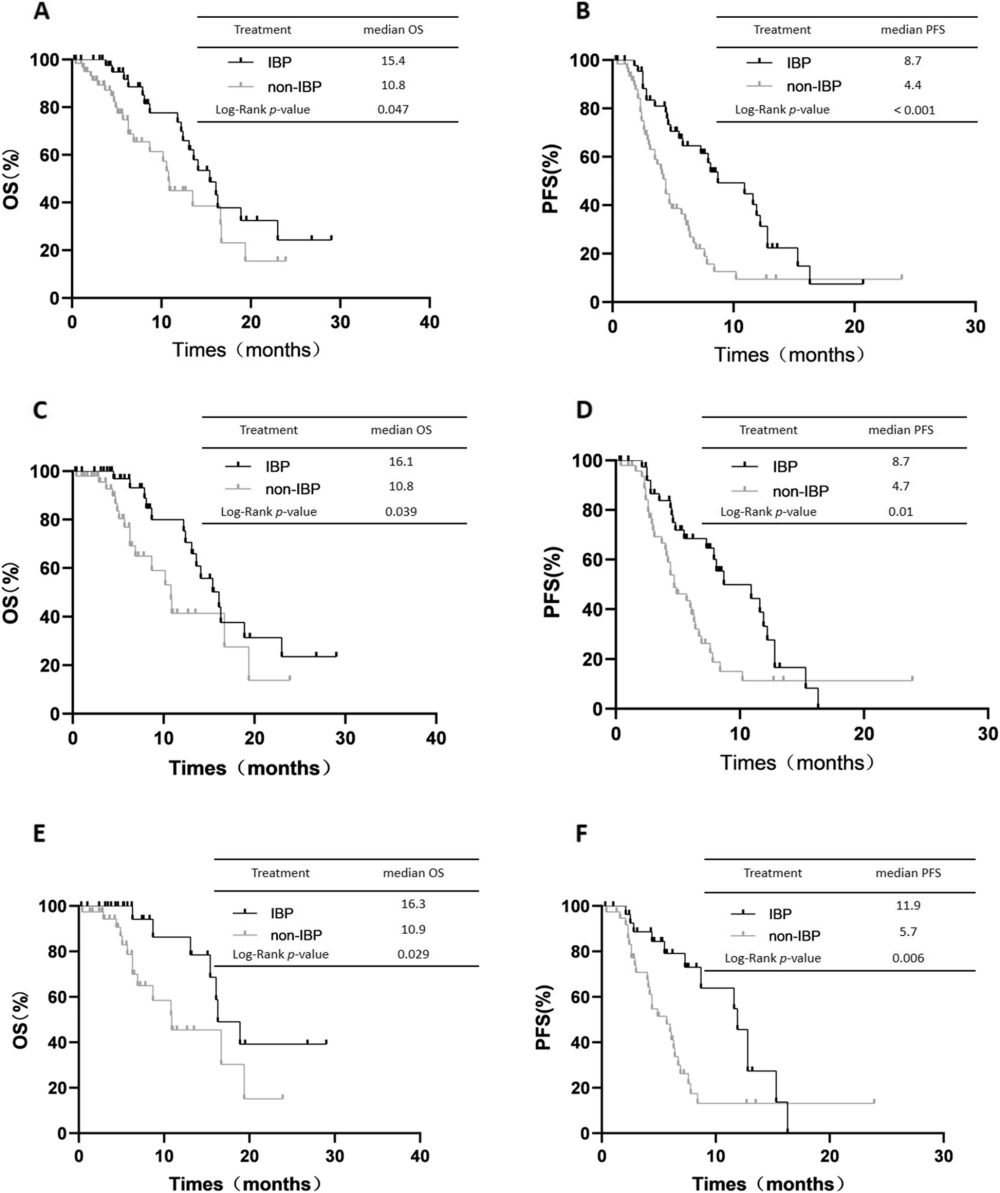
OS (**A**) and PFS (**B**) in patients with ≥4 cycles in first-line immunotherapy subgroup of aNSCLC between IBP and non-IBP. OS (**C**) and PFS (**D**) in patients with ≥6 cycles in first-line immunotherapy subgroup of aNSCLC between IBP and non-IBP. OS (**C**) and PFS (**D**) in patients with ≥8 cycles in first-line immunotherapy subgroup of aNSCLC between IBP and non-IBP

In the subgroup of 109 patients with ≥4 cycles in first-line immunotherapy, the OS and PFS were statistically different between the IBP group and non-IBP group (median OS: 15.4 vs. 10.8 months, respectively, *p*=0.047 Fig. 4A; median PFS: 8.7 vs. 4.4 months, respectively, *p*<0.001, Fig. 4B). And in the subgroup of the 90 patients with ≥6 cycles in first-line immunotherapy, the OS and PFS were statistically different between the IBP group and non-IBP group (median OS: 16.1 vs. 10.8 months, respectively, *p*=0.039, Fig. 4C; median PFS: 8.7 vs. 4.7 months, respectively, *p*=0.01, Fig. 4D). Then in the subgroup of the 67 patients with ≥8 cycles in first-line immunotherapy, the OS and PFS were statistically different between the IBP group and non-IBP group (median OS: 16.3 vs. 10.9 months, respectively, *p*=0.029, Fig. 4E; median PFS: 11.9 vs. 5.7 months, respectively, *p*=0.006, Fig. 4F).

**Fig. 5:**
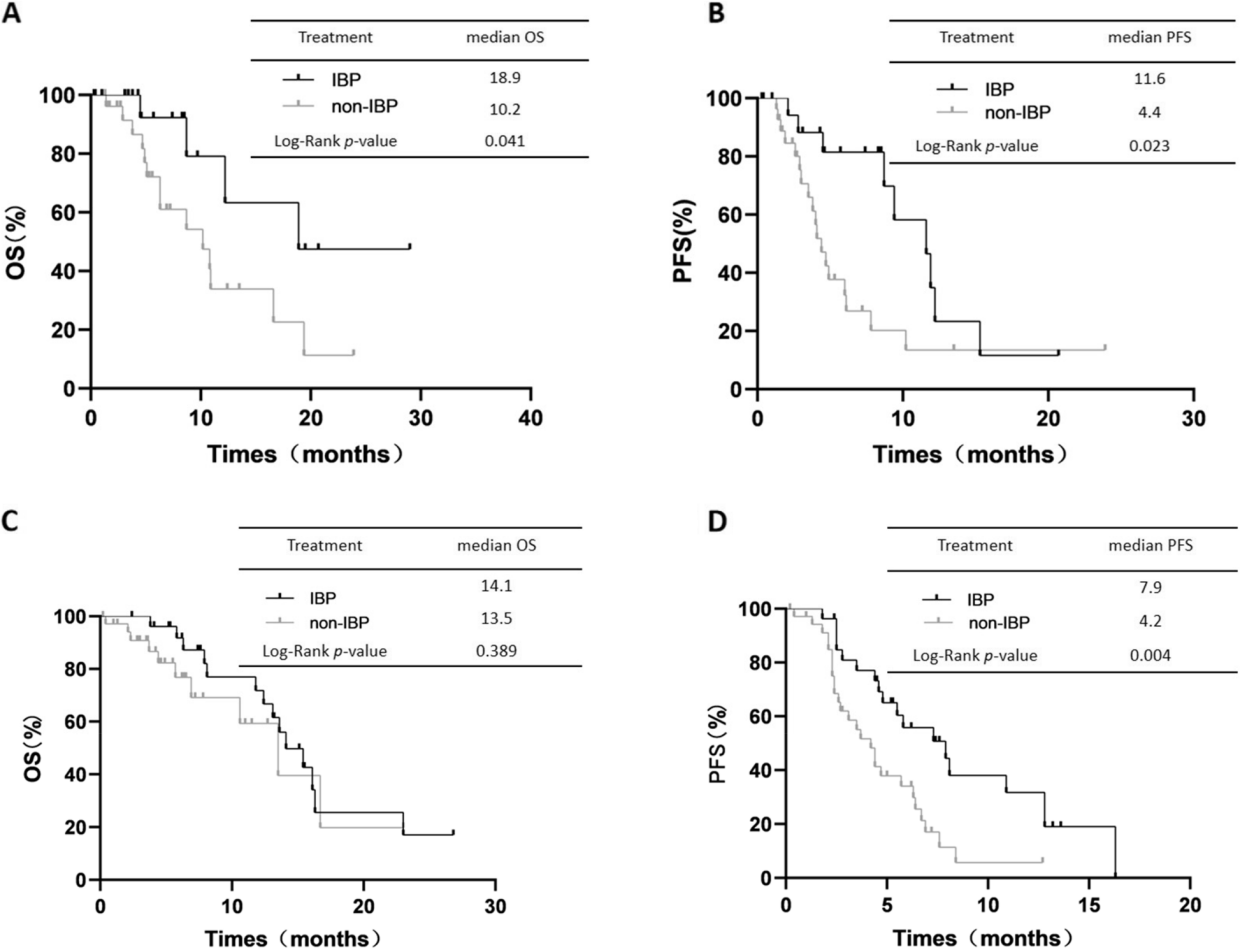
OS (**A**) and PFS (**B**) in patients with PR as best response in first-line immunotherapy subgroup of aNSCLC between IBP and non-IBP. OS (**C**) and PFS (**D**) in patients with SD as best response in first-line immunotherapy subgroup of aNSCLC between IBP and non-IBP

In the subgroup of the 48 patients with PR as best response in first-line immunotherapy, the OS was different between the IBP group and non-IBP group (median OS: 18.9 vs. 10.2 months, respectively, *p*=0.041, Fig. 5A). The PFS in the IBP group was longer than that in the non-IBP group (median PFS: 11.6 vs. 4.4 months, respectively, *p*=0.023, Fig. 5B). The OS of the 63 patients with SD as best response in first-line immunotherapy was similar between the IBP group and non-IBP group (median OS: 14.1 vs. 13.5 months, *p*=0.389, Fig. 5C). The PFS in the IBP group was longer than that in the non-IBP group (median PFS: 7.9 vs. 4.2 months, *p*=0.004, Fig. 5D).

**Fig. 6:**
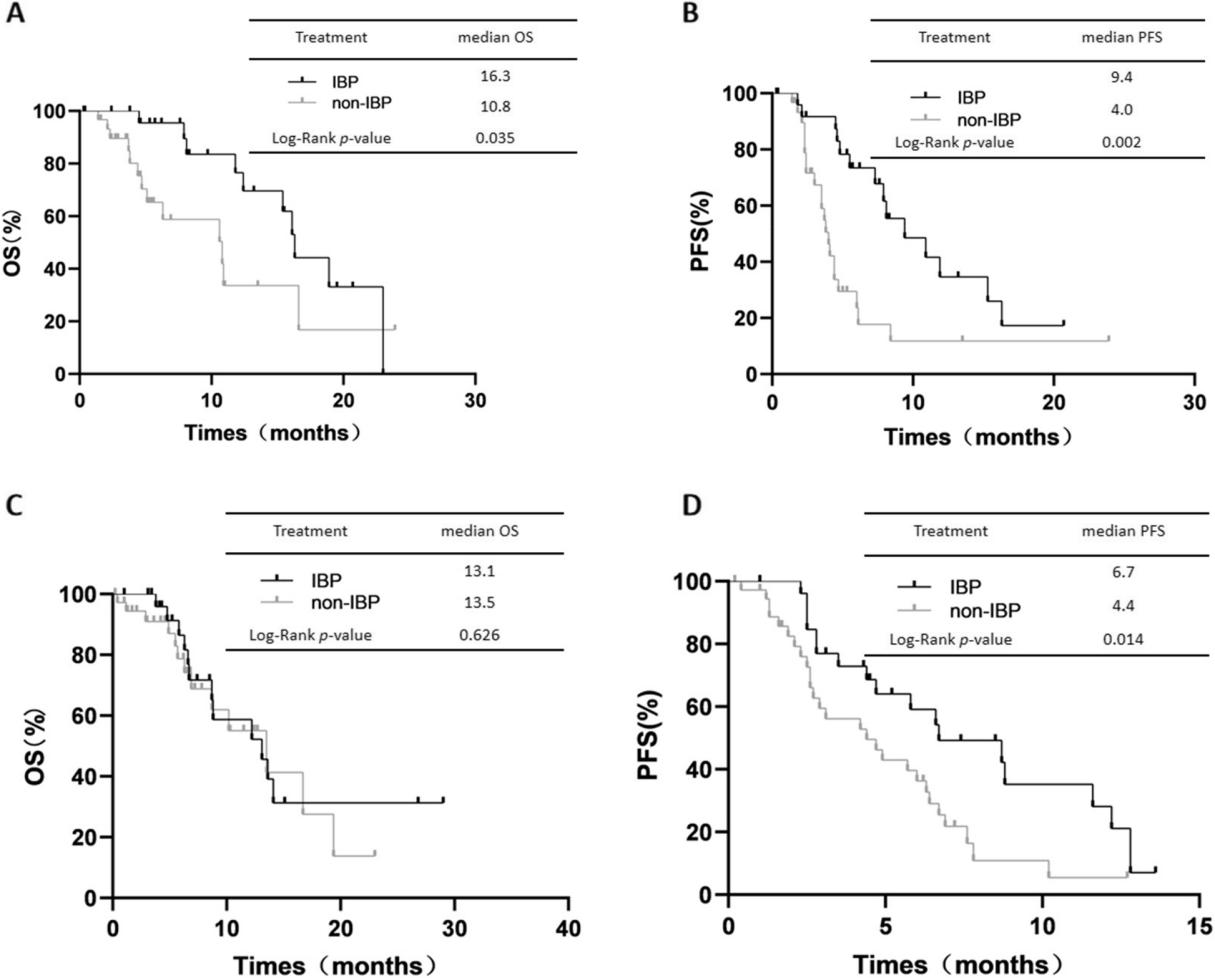
OS (**A**) and PFS (**B**) in patients with oligo progression in first-line immunotherapy subgroup of aNSCLC between IBP and non-IBP. OS (**C**) and PFS (**D**) in patients with extensive progrssion in first-line immunotherapy subgroup of aNSCLC between IBP and non-IBP

The IBP group showed a longer OS (median OS: 16.3 vs. 10.8 months, respectively, *p*=0.035, Fig. 6A) and PFS (median PFS: 9.4 vs. 4.0 months, respectively, *p*=0.002, Fig. 6B) than the non-IBP group for oligo progression subgroups. However, the OS (median OS: 13.1 vs. 13.5 months, respectively, *p*= 0.626, Fig. 6C) were not different between the two groups in the extensive progression subgroups. The PFS was longer in the IBP group than in the non-IBP group (median PFS: 6.7 vs. 4.4 months, respectively, *p*= 0.014, Fig. 6D).

### Prognostic factors

The clinical characteristics of the patients were evaluated to determine their prognostic value for OS (Table 2). Univariate analysis indicated that age and cycles of first-line immunotherapy were associated with survival. Patients ≤65 (*p*=0.045) and first-line immunotherapy ≥8 cycles (*p*=0.046) had a better OS. For PFS, univariate analysis revealed that histology, liver meta and cycles of first-line immunotherapy were significant favorable prognostic factors (Table 3). Multivariate analysis revealed that the liver meta (*p*= 0.007) was favorable prognostic factors for PFS.

**Table 2:** Univariate and multivariate analysis of OS

Factors	Univariate analysis of OS		Multivariate analysis of OS	
	HR(95%CI)	*p*	HR(95%CI)	*p*
Gender (male/female)	0.643(0.342-1.210)	0.71		
Age (<65/≥65)	1.790(1.013-3.163)	0.045	1.656(0.931-2.946)	0.086
Smoking status (never/smoking)	1.470(0.826-2.617)	0.190		
Histology(adenocarcinoma/squamous**)**	1.761(0.980-3.167)	0.059		
Brain meta (no/yes)	1.016(0.544-1.897)	0.960		
Liver meta (no/yes)	1.762(0.887-3.499)	0.106		
First-line cycles of immunotherapy (<4/≥4)	0.610(0.255-1.460)	0.267		
First-line cycles of immunotherapy (<6/≥6)	0.661(0.362-1.206)	0.177		
First-line cycles of immunotherapy (<8/≥8)	0.558(0.314-0.989)	0.046	0.603(0.338-1.075)	0.086
Best response to first line(PR/SD+PD)	1.057(0.586-1.906)	0.854		
Progressive mode(Oligo/Extensive)	1.013(0.573-1.791)	0.964

**Table 3:** Univariate and multivariate analysis of PFS

Factors	Univariate analysis of PFS		Multivariate analysis of PFS	
	HR(95%CI)	*p*	HR(95%CI)	*p*
Gender (male/female)	0.819(0.513-1.308)	0.403		
Age (<65/≥65)	0.994(0.634-1.559)	0.980		
Smoking status (never/smoking)	1.028(0.660-1.601)	0.903		
Histology(adenocarcinoma/squamous**)**	1.622(1.015-2.591)	0.043	1.345(0.831-2.177)	0.228
Brain meta (no/yes)	1.103(0.684-1.778)	0.689		
Liver meta (no/yes)	2.514(1.447-4.366)	0.001	2.186(1.242-3.844)	0.007
First-line cycles of immunotherapy (<4/≥4)	0.818(0.432-1.549)	0.538		
First-line cycles of immunotherapy (<6/≥6)	0.640(0.396-1.036)	0.070		
First-line cycles of immunotherapy (<8/≥8)	0.614(0.397-0.950)	0.029	0.701(0.448-1.097)	0.120
Best response to first line(PR/SD+PD)	1.348(0.851-2.137)	0.203		
Progressive mode(Oligo/Extensive)	1.336(0.852-2.095)	0.206		

### Toxicites

The rate of adverse events(AEs) in two groups was listed in Table 4. The incidence of any grade AEs in IBP group and non-IBP group was 32.1% and 29.4%, respectively. The rate of grade≥3 adverse events was 7.5% in the IBP group, compared to 5.9% in the non-IBP group. No patient died from AEs.

**Table 4:** Incidence of AEs

Treatment-related AEs, n(%)	IBP (*n*=53)	Non-IBP (*n*=68)
Any grade	17(32.1%)	20(29.4%)
Decreased neutrophil	6(11.3%)	7(10.3%)
Diarrhea	1(1.9%)	2(2.9%)
Anemia	2(3.8%)	1(1.6%)
Vomiting	1(1.9%)	2(2.9%)
Fatigue	1(1.9%)	1(1.5%)
Pneumonitis	2(3.8%)	1(1.5%)
Increased aspartate aminotransferase	1(1.9%)	2(2.9%)
Hypothyroidism	1(1.9%)	1(1.5%)
Myocarditis	0(0%)	1(1.5%)
Rash	1(1.7%)	0(0%)
Hypolbuminemia	1(1.9%)	2(2.9%)
Grade≥3	4(7.5%)	4(5.9%)
Decreased neutrophil	2(3.8%)	3(4.4%)
Diarrhea	2(3.8%)	1(1.5%)
AEs leading to discontinuation	2(3.8%)	2(2.9%)
Diarrhea	1(1.9%)	0(0%)
Decreased neutrophil	1(1.9%)	2(2.9%)
AEs leading to death	0(0%)	0(0%)

## Discussion

The available retrospective studies showed inconsistent clinical results. According to Enomoto et al, no significant benefits were associated with continuation of nivolumab for advanced NSCLC patients [11]. And two small sample studies reported no benefit from similar ICI rechallenge [12, 13]. However, several studies showed benefit in IBP group. A real-world study of more than 4,000 aNSCLC patients from the USA showed IBP patients had a longer OS (11.5 vs. 5.1 months *p*< 0.001) in comparison to non-IBP patients [14]. A multicenter study from Italy reported patients treated with nivolumab monotherapy as a second or subsequent line received longer OS (17.8 vs. 3.7 months *p*<0.0001) than not treated with nivolumab monotherapy beyond progression (NTBP)^15^. And a study of 125 aNSCLC patients by Ge et al. reported longer OS (26.6 vs. 9.5 months *p*< 0.001) in the IBP group [16]. Another research by Tian et al. also reported longer OS (15.7 vs. 5.0 months *p*< 0.001) in the IBP group [17]. Overall, the role of immunotherapy with ICI TBP in patients with NSCLC remains incompletely elucidated. In our study, there was no statistical difference of OS between two groups but results showed longer OS of IBP group than non-IBP group (14.1m vs 10.8m *p*=0.063). And IBP significantly prolonged PFS (8.7m vs 4.1m *p*<0.001). Previous studies included patients with driver genes and IBP >2 lines of therapy in their inclusion may explain the gap with our study in OS. In addition, previous studies used immune monotherapy as an option for immune continuation therapy, yet our immune monotherapy accounts for only 1.7%. The duration of first-line immunotherapy has always been a controversial issue due to the special mechanism of immunotherapy. In our study univariate analysis of OS and PFS suggested that first-line immunotherapy over 8 cycles was a favorable factor. According to the subgroup of first-line immunotherapy cycles analysis the OS and PFS were statistically different between the IBP group and non-IBP group in patients with ≥4 cycles and ≥6 cycles in first-line immunotherapy. Consistent with univariate analysis, patients with ≥8 cycles in first-line immunotherapy received longer OS (16.3m) and PFS (10.9m) than ≥4 cycles and ≥6 cycles. Our forest maps also show this trend of longer first-line cycles availability and longer survival. Lu Shun et al. presented their results about an exploratory research of response characteristics of the RATIONALE 304 study at the 2022 Chinese Society of Clinical Oncology (CSCO) meeting. Of 128 non-squamous NSCLC patients, 65 (50.8%) achieved first remission after 2 cycles 40 (31.3%) after 4 cycles and 100% after > 4 cycles [18]. Among 76 responder patients in a retrospective study of 262 patients (all cancer types) treated with an anti-PD-L1 monotherapy in a phase 1 trial, the median time from therapy initiation to response was 2 months [19]. But 28 responder patients responsed until 3 months later. Our study included patients treated with ≥2 cycles (about 2 months) in first-line and results, while patients with ≥4 cycles (about 3 months) received longer survival. And our results suggested that the longer first-line cycles the longer survival. Studies reported that the incidence of pseudoprogression in non-small cell lung cancer was about 5.4% [20]. And approximately 2.2% of patients assessed as progressing under traditional RECIST 1.1 criteria had a CR or PR as measured by iRECIST(immune-related RECIST) [21]. Different from RECIST 1.1, iRECIST defines the PD determined by RECIST 1.1 as immune unconfirmed progressive disease (iUPD), and re-evaluates after 4-8 weeks. Then the next evaluation confirms progress as immnue confirmed progressive disease (iCPD) or confirms as iCR, iPR, iSD for next assessment [35]. Patients with ≥4 cycles of therapy may have overcome atypical responses such as pseudoprogression and are more likely to benefit from immunotherapy. The statistical difference of ≥4 cycles suggested that the first-line immunization with 4 cycles before imaging efficacy assessment may be more consistent with the actual disease status of patients with antiimmunotherapy.

The effect of best response of initial immunotherapy on survival has been explored in studies. Ge et al's findings showed that IBP patients had longer OS and PFS than non-IBP patients, whether in the subset of patients who responded to the initial immunotherapy or in those who did not [16]. In a similar vein, Ricciuti et al. observed a survival benefit for patients in the IBP group compared to non-IBP patients, independent of the best response to the initial immunotherapy, whether disease control or PD [15]. This was based on a subgroup analysis of IBP with nivolumab. Both studies included patients with multiple lines of IBP as well as a subgroup of patients with EGFR mutation. However, in previous studies, immunotherapy had a shorter OS in EGFR-positive patients and had an increased risk of interstitial pneumonia [2, 22, 23]. Interestingly patients who had a positive response to earlier immunotherapy had greater survival benefit than those who had a negative response as best response. The mPFS of patients in (CR/PR) group verus (SD/PD) group was 7.3m verus 4.3m (*P*<0.0001), and mOS of these patients was 22.8m verus 15.7m (*P*<0.0001). The ORR for patients who experienced the best response to the first round of ICI treatment—CR/PR/SD/PD was 100%, 6.7%, 10.1%, and 10.2%, respectively [17]. The same phenomenon was observed in melanoma [24, 25]. Our results showd IBP benefit in both OS (18.9 vs. 10.2 months, *p*=0.041) and PFS (11.6 vs. 4.4 months, *p*=0.023) in the PR as best response in first-line immunotherapy subgroup. Patients who responded well to prior immunotherapy received increased survival benefit from the second-line ICI-based treatment. Although the exact mechanism behind this finding is unclear, one possible explanation could be that patients who responded well to previous immunotherapy produced immune memory cells [26, 27], thereby rapidly rebuilding the immune system during the next round of immunotherapy.

The clinical concept of OPD (oligo progression disease) was first introduced in 2011 in order to differentiate the degree of progression to identify patients with potentially manageable progression [28]. OPD occurs after an initial response to systemic therapy and anatomically limited tumor progression in otherwise controllable. In previous studies, OPD mostly described aNSCLC with driver genes. The OPD rate of patients with targeted therapy was 33%-72% [29–31]. However, in the studies of immunotherapy, OPD rate of patients is reported to be lower as 10%-55.3% [32, 33]. Consistent with previous studies, the OPD rate in our study for first-line immunotherapy was 47.1% (57/121), slightly less than extensive progression. In addition, patients in the OPD subgroup achieved longer OS (16.3 vs 10.8 monthes *p*=0.035) and PFS (9.4 vs 4.0 monthes *p*=0.002) with continued immunotherapy after progression. The best management for OPD patients remains unclear cause lack of published prospective data available. Previous studies suggest that the addition of local therapy and maintenance of the original systemic therapy regimen is feasible to regain control of disseminated tumors [32, 33]. They reported the benefit of adding local radiation therapy in combination with immunotherapy. Since only 23% (6/26) of patients in our IBP group added topical treatment, no further analysis was performed. Local radiation therapy can enhance the immunostimulatory effect and allow systemic therapy to continue by overcoming the few subclones that develop resistance [34] and is increasingly seen as a promising combination treatment strategy with ICIS. However, the optimal dose of radiation therapy to induce immune stimulation and the appropriate sequence of treatment (sequential or concurrent) were needed further prospective studies are needed to further elucidate.

Like all retrospective analyses, our study has limitations. First, the small sample size affected the statistical power and may have led to selection and measurement bias. Despite adjustment by the Cox regression model, confounding factors may still have been present. Further analysis with a larger sample size is necessary in the future. Due to the moderate sample size, we did not differentiate and assess the efficacy of each treatment regimen in the combination group, and this issue requires more research. More potential influencing factors, such as smoking [36] and immunotherapy concomitant drugs [37], also need more detailed data for further study. And These results will need to be confirmed by prospective randomized studies in sizable populations.

## Conclusion

In conclusion, the clinical outcomes of IBP were similar to those of non-IBP in patients with PD after first-line immnuotherapy in advanced NSCLC. But IBP is an effective therapy options for patients who with ≥4 cycles immunotherapy PR as best response or oligoprogression in first-line. Our observations may provide direction for treatment options for patients after progression of first-line immunotherapy. Larger trials are needed for further confirmation.

## Data Availability

The datasets used and/or analysed during the current study are available from the corresponding author on reasonable request.

## Abbreviations

NSCLC: Non-small cell lung cancer;
ICI: Immune checkpoint inhibitor
IBP: Immunotherapy beyond progression
PD-1: Anti-programmed cell death-1
PD-L1: Anti-programmed cell death ligand-1
PD: Progressive disease
CT: Chest computed tomography
PET: Positron emission tomography
MRI: Magnetic resonance imaging
RECIST: Response Evaluation Criteria in Solid Tumors
AEs: Adverse events
CTCAE: Common Terminology Criteria for Adverse Events
OS: Overall survival
PFS: Progression-free survival
